# Staying Relevant in the Digital Age: Exploring the Evolving Frontier of Telehealth for Mental Health in the Military Health System and Veterans Health Administration

**DOI:** 10.1007/s11920-025-01651-3

**Published:** 2025-12-29

**Authors:** Elizabeth A. Greene, Eric J. Serpico, Gary L. Legault, Scott G. Williams

**Affiliations:** Uniformed Services University of the Health Sciences, Bethesda, MD USA

**Keywords:** Military, Veteran, Telehealth, Telemedicine, Psychotherapy, Mental health

## Abstract

**Purpose of Review:**

This review examines recent evidence for the effectiveness of telehealth in treating Post-Traumatic Stress Disorder (PTSD), Major Depressive Disorder (MDD), alcohol use disorder, and insomnia in military veterans and active-duty service members (ADSMs).

**Recent Findings:**

Recent randomized controlled trials and prospective cohort studies provide strong evidence that synchronous video-teleconference based therapy is effective for PTSD, MDD, and insomnia in this population. There is growing evidence for the effectiveness of internet-based self-guided therapy, particularly when combined with coaching support provided by telemedicine, for symptoms of PTSD, MDD, and insomnia. The effectiveness of telehealth in the treatment of alcohol use disorders is less well-supported, as is the effectiveness of mobile applications. These findings are supported by the team’s analysis of the literature as well as an analysis provided by an Artificial Intelligence (AI) platform.

**Summary:**

The current evidence supports the use of synchronous video-teleconference and internet-based self-guided therapy with coaching support in the treatment of several common diagnoses in the military veteran and ADSM populations. Other modalities of telehealth require further research.

**Human and Animal Rights:**

This article does not contain any studies with human or animal subjects performed by any of the authors.

**Supplementary Information:**

The online version contains supplementary material available at 10.1007/s11920-025-01651-3.

## Introduction

Telehealth, defined as the provision of healthcare across a geographic distance enabled by information and telecommunication technologies, has long been important in both the Military Health System (MHS) and Veterans Health Administration (VHA) [1–3]. These two systems provide care for individuals across vast geographic distances, and telehealth offers the promise of sharing resources and expertise across that network. Across the field, definitions for telehealth continue to vary; in this article the term telehealth encompasses telemedicine (medical care provided via synchronous video-teleconference (VTC) or telephone), consultation with remote medical specialists synchronously or asynchronously, technology to monitor patients remotely, collection of patient data through wearable devices, use of internet-based platforms to provide care, use of mobile applications, and use of artificial intelligence to advance patient care [4]. 

The MHS and VHA have considered various approaches for mental telehealth care for many years [2, 5]. The VHA has been a pioneer in telehealth, with ongoing robust telehealth services starting in 2010 and further significant expansion during the COVID-19 pandemic [1]. Benefits of telehealth for the VHA include expanded access for veterans living in rural areas due to decreased travel time and reduction of the physical burden of appointments for those with disabilities [6]. The MHS began utilizing telehealth in the 1990’s to support servicemembers in deployed locations, was slower to adopt telehealth for non-deployed servicemembers and family members, and then experienced a 20-fold increase in telehealth visits during the COVID-19 pandemic [7]. In the MHS, telehealth offers the opportunity to improve care for deployed servicemembers, reduce stigma, and expand access to care for both servicemembers and family members [8]. The use of telehealth for mental health care increased rapidly in both systems during the COVID-19 pandemic and has continued at higher levels than were seen prior to the pandemic [9–11]. A study by the RAND corporation on MHS staff perspectives on the use of telehealth during the pandemic noted that staff were using telehealth, although with some concerns and the desire for more education and support [12]. A recent study noted that about half of veterans express a preference for telehealth treatment, citing flexibility and increased access to care [13]. 

Early analyses of the treatment of Post-Traumatic Stress Disorder (PTSD) and Major Depressive Disorder (MDD) via telehealth suggested that telemedicine-based treatment was effective for both conditions [14, 15]. This finding has continued to be supported in more recent reviews and studies, including two reviews that found synchronous telemedicine non-inferior to in-person care for mental health conditions, and a large retrospective record review of active duty service members and veterans receiving care in a network outside the VHA and MHS which showed telemedicine had small but significant advantages over in-person care for PTSD, MDD and Generalized Anxiety Disorder [16–18]. Internet based psychotherapy has also been examined for effectiveness, with two reviews that suggested potential benefit although the authors cautioned that the included studies were heterogenous and recommended additional research [19, 20]. 

Several of the authors (EG, ES and SW) are psychiatrists within or retired from the MHS, with a collective 55 years of experience providing mental health care to active duty servicemembers (ADSMs), Veterans, and family members. One author (GL) directs the MHS’s Virtual Medical Center, including a virtual mental health clinic. Effective, accessible treatments for common conditions such as PTSD, MDD, Insomnia, Substance Use Disorders (SUD), Anxiety Disorders and Adjustment Disorders are more than theoretical concerns for us; these are urgent issues that directly impact the people we care for. Effective virtual mental health care is suggested as part of the approach to improving mental health care in the military health system [10]. As such, we were interested in the current evidence for the effectiveness of telehealth treatment for conditions we see every day in our clinical work with ADSMs and Veterans, particularly in the post-pandemic period. We wanted to examine evidence not only for synchronous telemedicine but also for internet-based mental health care and mobile application-based health care. We also wondered how emerging AI platforms might contribute to synthesizing medical literature to answer a clinical question.

## Methods

As a team, we formulated our core research question: “What is the evidence over the past five years for the benefit of telehealth treatment for military and veteran populations with PTSD, MDD, Insomnia, SUD, Anxiety Disorders or Adjustment Disorders?” To answer this question we searched PubMed, Embase and PsycINFO using variants of the terms tele-behavioral health, tele-mental health, telepsychiatry, telepsychology, veteran, military, post-traumatic stress disorder, major depressive disorder, substance use disorder, anxiety disorder, and adjustment disorder for articles published between 2019 and 2025. The search was initially conducted in September 2024, and a repeat search was conducted in May 2025 to identify articles that had been published in the interval.

**Fig. 1:**
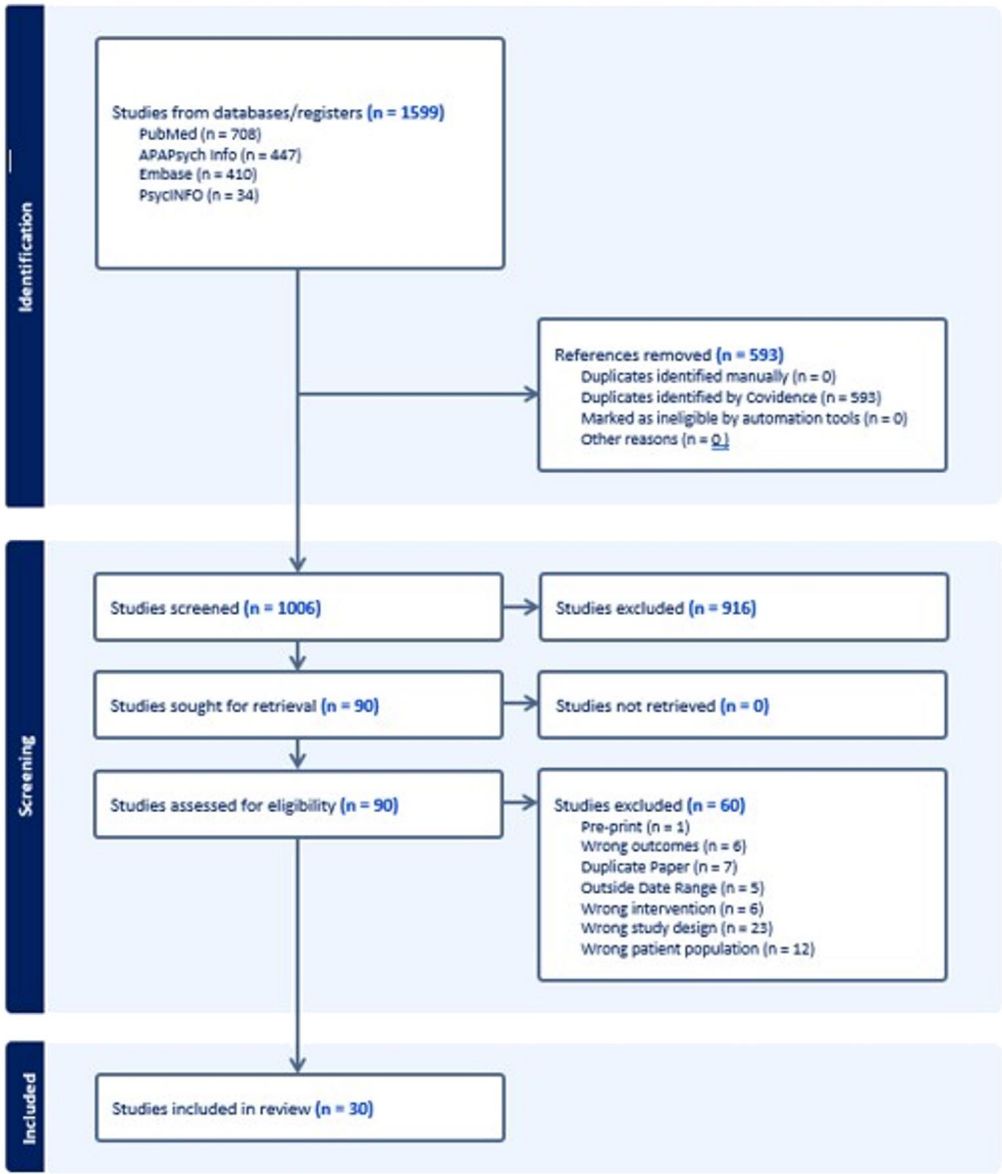
PRISMA Diagram

Included articles were prospective cohort or randomized controlled trials of telehealth in military or veteran populations and which had clinical outcomes with any validated clinical scale. We restricted the focus of the search to treatment to PTSD, MDD, Insomnia, SUD, Anxiety Disorders and Adjustment Disorders. Telehealth was considered broadly to include synchronous video teleconferencing, internet-based psychotherapy, and treatment delivered by mobile applications. We excluded studies that only reported feasibility or acceptability outcomes, retrospective record reviews, case reports, populations that were not military or veteran, and diagnoses of pain, traumatic brain injury, personality disorders, neurodevelopmental disorders, bipolar disorder, or schizophrenia. We also excluded secondary analyses of data sets that were represented by a primary publication. We utilized Covidence™ (Melbourne, Australia) as a platform for article screening and full text review. All articles were screened by two reviewers. Full text review was also completed by two reviewers. We identified thirty articles that met the specified inclusion criteria. Data extraction was conducted by one team member (EG) based on a data extraction template agreed on by all team members and was reviewed and discussed as a team. The flow of articles through the process is depicted in this PRISMA diagram (Fig. 1).

### Studies Identified

Thirty papers met our inclusion criteria; one of these papers [21] reported data from two studies with different methodologies and is counted twice so that each data set is represented. All of them focused on the provision of psychotherapy via telehealth, rather than medication management. These studies involved 3,379 participants; 2,486 of these were participants in randomized controlled trials. All studies included veteran populations, with 9.7% also including active-duty military members. Most studies were conducted in the United States (87.1%) with a few studies occurring in the United Kingdom and in Germany. Telehealth modalities included synchronous VTC (38.7%), internet-based self-guided therapy (48.4%) and mobile application-based therapy (12.9%). Most studies examined PTSD (77.4%); also studied were participants with significant symptoms of depression, hazardous alcohol use, and insomnia. We did not find studies focused on Anxiety Disorders, Adjustment Disorders, or substance use disorders other than hazardous alcohol use that met our inclusion criteria.

### Synchronous Video-Teleconference

Seven studies compared therapy delivered by VTC directly to in-person therapy, and five additional studies reported the outcomes of VTC therapy. All but two of these studies focused on the diagnosis of PTSD; Insomnia and symptoms of depression in Parkinson’s Disease were the focus of the other two studies. A summary of these studies is presented in Table 1. Overall, VTC based therapy was either non-inferior or superior to in-person therapy in direct comparison, which is consistent with prior studies and analyses noted in the introduction. Studies comparing different types of therapy conducted by VTC found benefit, as did prospective cohort studies of VTC therapy. Of particular interest were the two studies that compared in-home telehealth to in-home in-person care and office-based care, which found that in-home telehealth had similar improvements in symptoms to the other modalities.[24, 30] Group therapy and couple therapy were also represented in these studies, and although these studies were less robust, they suggest that telehealth can extend to these forms of therapy and merits further research [22, 29, 31].

**Table 1:** Studies of telehealth using synchronous video-teleconference

Citation	Veteran or Active Duty	Telehealth Modality	Control	Program Name	Primary Diagnosis	Study Type	Number of Subjects	Primary Outcome Measure	Result Summary
Glassman et al., 2019[22]	Veteran	synchronous VTC - group and individual	In person group and individual	Cognitive Processing Therapy	Post-Traumatic Stress Disorder	randomized controlled trial	251	Quality of Life Inventory (QOLI)	Quality of Life improved, no difference between in person or VTC treatment
Liu et al., 2020[23]	Veteran	synchronous VTC - individual	in person individual	Cognitive Processing Therapy	Post-Traumatic Stress Disorder	randomized controlled trial	207	Clinician Administered PTSD Scale (CAPS)	Both groups improved; improvement inferior in VTC group post-treatment, but no between group differences at 6-month follow-up
Morland et al., 2020[24]	Veteran	synchronous VTC - individual (home based)	In home in person therapy; synchronous VTC - individual (office-based)	Prolonged Exposure Therapy	Post-Traumatic Stress Disorder	randomized controlled trial	175	Clinician Administered PTSD Scale for DSM-5 (CAPS5)	Post-Traumatic Stress Disorder symptoms improved in all groups, no statistically significant difference between groups. Office based treatment had the highest dropout rate.
Murphy & Turgoose, 2020[25]	Veteran	synchronous VTC - individual	none	Cognitive Processing Therapy	Post-Traumatic Stress Disorder	prospective cohort	27	PTSD Checklist for DSM-5 (PCL5)	Statistically significant improvement in Post-Traumatic Stress Disorder symptoms.
Acierno et al., 2021[26]	Veteran	synchronous VTC - individual	in person individual	Prolonged Exposure Therapy	Post-Traumatic Stress Disorder	randomized controlled trial	136	PTSD Checklist for DSM-5 (PCL-5)	Post-Traumatic Stress Disorder symptoms improved in all groups, no statistically significant difference between groups.
Dobkin et al., 2021[27]	Veteran	synchronous VTC - individual	Treatment as usual (no therapy intervention)	Cognitive Behavioral Therapy	Depression in Parkinson’s Disease	randomized controlled trial	90	Hamilton Depression Rating Scale (HAM-D)	Depression symptoms improved.
Reich et al., 2021[28]	Veteran	synchronous VTC - individual	in person individual	Prolonged Exposure Therapy	Post-Traumatic Stress Disorder	randomized controlled trial	150	Inventory of Psychosocial Functioning (IPF)	Improved interpersonal functioning was seen in both groups, no difference between groups.
Hendrickx et al., 2022[29]	Veteran	Synchronous Telemedicine (Video) - couple	none	Cognitive Behavioral Conjoint Therapy^a^	Post-Traumatic Stress Disorder	prospective cohort	6	PTSD Checklist for DSM-5 (PCL-5)	Improvement in PTSD symptoms
Peterson et al., 2022[30]	Veteran	synchronous VTC - individual	in home in person therapy; in office in person therapy	Cognitive Processing Therapy	Post-Traumatic Stress Disorder	randomized controlled trial	120	PTSD Checklist for DSM-5 (PCL-5) Clinician Administered PTSD Scale for DSM-5 (CAPS5)	in-home in person and home based teletherapy had statistically significantly better outcomes than office based in person care
Arizmendi et al., 2023[31]	Veteran	synchronous VTC - group	in person group	Cognitive Behavioral Therapy for insomnia	Insomnia	historically controlled trial	27	Insomnia Severity Index (ISI)	Telehealth similar to face to face treatment
LoSavio et al., 2023[32]	Veteran	synchronous VTC – individual and in-person	none	Written Exposure Therapy	Post-Traumatic Stress Disorder	prospective cohort	277	PTSD Checklist for DSM-5 (PCL-5)	Cohort included both VTC and in-person participants. Similar improvement in PCL-5 symptoms in in-person and telehealth delivery. Telehealth delivery resulted in higher completion rate.
Cloitre et al., 2024[33]	Veteran	synchronous VTC - individual	present centered therapy	STAIR^b^	Post-Traumatic Stress Disorder	randomized controlled trial	161	Clinician Administered PTSD Scale for DSM-5 (CAPS-5)	Both intervention and control delivered via VTC; STAIR participants had greater improvement than control group.

a. Cognitive Behavioral Conjoint Therapy – treatment focused on treating PTSD that involves the affected individual’s romantic partner

b. STAIR – Skills Training in Affective and Interpersonal Regulation – 12-week individual therapy focused on emotion management and interpersonal patterns and communication

### Internet-Based and Mobile Application

Fifteen studies examined the potential benefits of internet-based self-guided therapy, and four studies evaluated mobile applications providing therapy. These telehealth modalities offer the potential for asynchronous care that can be accessed at the convenience of patients, anytime and anywhere. We found studies investigating treatment for PTSD, depression, hazardous alcohol use, and insomnia. A summary of these studies is presented in Table 2. Fourteen of the fifteen studies on internet-based therapy showed benefit in symptom reduction; the one study that did not show benefit was a small prospective cohort study [36]. Nine of the studies showing benefit utilized coaching in addition to internet-based therapy. Coaching occurred through multiple modalities (text based asynchronous, phone calls, VTC, and in-person), by multiple types of support personnel (peer coaches, study-based coaches, and therapists), and at variable intervals (one coaching session per internet-based therapy session or one coaching session per every two internet-based therapy sessions). Identifying the optimal timing and types of support for patients using internet-based therapy is an intriguing way to maximize both benefit to patients and the use of the limited resource of therapist time.

**Table 2:** Studies of telehealth using Internet-based and mobile applications

Citation	Veteran or Active Duty	Telehealth Modality	Control	Program Name	Primary Diagnosis	Study Type	Number of Subjects	Primary Outcome Measure	Result Summary
Possemato et al., 2019[34]	Veteran	internet based self-guided therapy intervention with in person peer support	internet based self-guided therapy without peer support	Thinking Forward^a^	Alcohol Use and Post-Traumatic Stress Disorder	randomized controlled trial	30	PTSD Checklist - Military Version (PCL-M), Drinking Days (DD)	Improvement in Post-Traumatic Stress Disorder symptoms with or without peer support; no statistically significant change in Drinking Days
Livingston et al., 2020[35]	Veteran	Internet based self-guided therapy intervention	none	VetChange^b^	Alcohol Use	prospective cohort	222	Average Weekly Drinks (AWD)	Decrease in average weekly drinks
Niemeyer et al., 2020[36]	Both	internet based therapist guided therapy	none	Internet based Cognitive behavioral Therapy	Post-Traumatic Stress Disorder	prospective cohort	37	Clinician Administered PTSD Scale for DSM-5 (CAPS5)	No statistically significant improvement in Post-Traumatic Stress Disorder Symptoms.
Bauer et al., 2021[37]	Veteran	internet based self-guided therapy with VTC coaching by therapists	none	WebSTAIR^c^	Post-Traumatic Stress Disorder, Depression	prospective cohort	80	PTSD Checklist for DSM-5 (PCL5), Patient Health Questionnaire − 9 (PHQ9)	Both Post-Traumatic Stress Disorder and Depression symptoms improved.
Creech et al., 2021[38]	Veteran	internet based self-guided therapy	none	SHE^d^	Post-Traumatic Stress Disorder, Alcohol Use Disorder	prospective cohort	20	Alcohol Use Disorders Identification Test (AUDIT), PTSD Checklist for DSM-5 (PCL-5)	Post-Traumatic Stress Disorder symptoms improved by 4-month follow-up. No change in hazardous alcohol use.
Hermes et al., 2021[39]	Veteran	Internet based self-guided therapy	none	SHUTi^e^	Insomnia	prospective cohort	77	Insomnia Severity Index (ISI)	Insomnia symptoms improved.
Lehavot et al., 2021[40]	Veteran	internet based self-guided therapy with coaching phone calls from study coach	phone coaching without internet based self-guided therapy	DESTRESS-WV^f^	Post-Traumatic Stress Disorder	randomized controlled trial	102	PTSD Checklist for DSM-5 (PCL-5)	Post-Traumatic Stress Disorder symptoms improved in both groups, no difference between groups.
McLean et al., 2021[21]	Both	internet based self-guided therapy with asynchronous text based and synchronous phone call coaching	present centered therapy	WebPE^g^	Post-Traumatic Stress Disorder	randomized controlled trial	40	PTSD Checklist for DSM-5 (PCL-5)	Post-Traumatic Stress Disorder symptoms improved in both groups, no difference between groups.
McLean et al., 2021[21]	Both	internet based self-guided therapy with asynchronous text based and synchronous phone call coaching	none	WebPE	Post-Traumatic Stress Disorder	prospective cohort	34	PTSD Checklist for DSM-5 (PCL-5)	Post-Traumatic Stress Disorder symptoms improved.
Cloitre et al., 2022[41]	Veteran	internet based self-guided therapy with VTC coaching by therapists every other session	internet based self-guided therapy with VTC coaching by therapists every session	WebSTAIR	Post-Traumatic Stress Disorder, Depression	randomized controlled trial	202	PTSD Checklist for DSM-5 (PCL-5), Patient Health Questionnaire − 9 (PHQ9)	Post-Traumatic Stress Disorder, Depression symptoms improved in both groups, no difference between groups.
Fletcher et al., 2022[42]	Veteran	internet based self-guided therapy plus VTC therapist coaching	none	WebSTAIR	Post-Traumatic Stress Disorder, Depression	prospective cohort	32	PTSD Checklist for DSM-5 (PCL5), Patient Health Questionnaire − 9 (PHQ9)	Improvement in Post-Traumatic Stress Disorder symptoms and in Depression symptoms.
Kuhn et al., 2022[43]	Veteran	mobile application	waitlist	Insomnia Coach^h^	Insomnia	randomized controlled trial	50	Insomnia Severity Scale (ISI), Patient Reported Outcomes Measurement Information System - Sleep Related Impairment (PROMIS-SRI)	Improvement in insomnia symptoms was significantly better in intervention than in waitlist control group.
Leightley et al., 2022[44]	Veteran	mobile application	mobile application that provided information only	Drinks: Ration ^i^	Alcohol Use	randomized controlled trial	123	Timeline Follow-Back for Alcohol Consumption (TLFB); Alcohol Use Disorder Identification Test (AUDIT)	Reduction in alcohol use seen in active intervention at primary endpoint, but by 168-day follow-up no between group differences.
McLean et al., 2022[45]	Veteran	mobile application	waitlist	Renew^j^	Post-Traumatic Stress Disorder	randomized controlled trial	93	PTSD Checklist for DSM-5 (PCL-5)	No difference between groups for change in PCL-5
Nazem et al., 2023[46]	Veteran	Internet based self-guided therapy	insomnia education website	SHUTi	Insomnia	randomized controlled trial	231	Insomnia Severity Index (ISI)	SHUTi participants had statistically significant greater improvement than control group.
Kim et al., 2024[47]	Veteran	internet based self-guided therapy intervention plus biweekly synchronous VTC group therapy	none	WebSTAIR	Post-Traumatic Stress Disorder, Depression	prospective cohort	39	PTSD Checklist for DSM-5 (PCL5), Patient Health Questionnaire − 9 (PHQ9)	Participants experienced decreases in both PCL-5 and PHQ-9.
McLean et al., 2024[48]	Veteran	internet based self-guided therapy intervention: Written exposure plus online chats vis instant messaging before and after exposure with a peer coach	internet based self-guided therapy intervention: Verbal exposure plus online chats vis instant messaging before and after exposure with a peer coach	Written Exposure Therapy	Post-Traumatic Stress Disorder	randomized controlled trial	124	PTSD Checklist for DSM-5 (PCL-5), Patient Health Questionnaire − 9 (PHQ9)	Both intervention and control delivered via internet based self-guided therapy plus coaching with online chat. Both groups experienced significant reductions in PCL-5 and PHQ-9 scores.
Morland et al., 2024[49]	Veteran	internet based self-guided therapy plus phone coaching by therapists	none	Couple HOPES^k^	Post-Traumatic Stress Disorder	prospective cohort	15	PTSD Checklist for DSM-5 (PCL-5), Collateral PTSD Checklist for DSM-5 (cPCL-5)	Participants experienced a reduction in PCL-5 scores and partners reported similar reduction on the cPCL-5.
Davis et al., 2025[50]	Veteran	mobile application	stress management education delivered via internet	Mind Guide^l^	Post-Traumatic Stress Disorder and Alcohol Use Disorder	randomized controlled trial	201	PTSD Checklist for DSM-5 (PCL-5), Drinking Days (DD)	Mind Guide participants experienced a significant reduction in PTSD symptoms compared to control group. No significant difference between group in drinking days.

a. Thinking Forward – 12-week structured cognitive behavioral program targeting hazardous alcohol use

b. VetChange – web-based application for Veterans with hazardous alcohol use and PTSD symptoms

c. WebSTAIR – 10-session web-based transdiagnostic intervention for trauma exposed individuals adapted from Skills Training for Affective and Interpersonal Regulation

d. SHE – Safe and Healthy Experiences; a motivational interviewing intervention with modules for PTSD, hazardous alcohol use, and interpersonal violence

e. SHUTi – Sleep Healthy Using The Internet; a 6-session self-guided cognitive behavioral therapy based program which is interactive and tailored to the individual

f. DESTRESS-WV – Delivery of Self-Training and Education for Stressful Situations – Women Veterans; online modules include coping skills, written exposure, and relapse prevention

g. WebPE – Web based delivery of Prolonged Exposure; a 10-session self-guided program following the Prolonged Exposure protocol, with asynchronous therapist feedback

h. Insomnia Coach – mobile application delivering stimulus control, sleep restriction, cognitive restructuring, relaxation training and sleep psychoeducation

i. Drinks:Ration – mobile application delivering interactive education, individualized guidance and goal setting, and monitoring of alcohol use

j. Renew – mobile application for PTSD; exposure based self-management with an integrated peer support function

k. Couple HOPES – Couple Helping Overcome PTSD and Enhance Satisfaction; a 7-session Cognitive Behavioral Conjoint Therapy web-based application

l. Mind Guide – mobile application with 16 modules on mindfulness coaching and mindfulness based relapse prevention

Of particular interest was the online program webSTAIR (web Skills Training in Affective and Interpersonal Regulation); four different studies were conducted on this intervention using different frequencies and formats of coaching, and benefit was found in all four studies for symptoms of PTSD and depression [37, 41, 42, 47]. This program is currently available free of charge from the National Center for PTSD. Another intervention that showed benefit in multiple studies was SHUTi (Sleep Healthy Using the Internet); two studies (one prospective cohort and one randomized controlled trial) showed benefit and were conducted without coaching [39, 46]. This program is currently available for clinical trials and studies from the University of Virginia.

Studies on mobile applications were more divided in their results; the application Insomnia Coach (for insomnia symptoms) showed benefit when compared to a waitlist control [43]. The application Mind Guide (for PTSD and hazardous alcohol use symptoms) showed benefit in reducing PTSD symptoms when compared to a stress management psychoeducational application but no improvement in hazardous alcohol use [50]. The app Drinks: Ration showed short term benefit in reduced alcohol use but benefit was not sustained in longer-term follow-up [44]. The application Renew, an application for PTSD symptoms using an exposure based self-management strategy, did not show improvement in PTSD symptoms compared to a waitlist control [45]. All of the studies on mobile applications took place since 2022, suggesting this is an area of research that is still developing. A suite of mobile applications has been developed by the Department of Defense and the Veterans Health Administration; these are available to users free of charge at https://mobile.va.gov/ and https://mobile.health.mil/. While the evidence does not yet support the use of mobile applications as sole interventions, these free applications seem to be low risk and may serve as adjuncts to other forms of care.

### Limitations

Telehealth offers the promise of expanded access to high-quality, effective mental health care for patients in the MHS and VHA, but ongoing research is needed. Telehealth is a potentially powerful tool for mental health, but its limitations, including challenges in managing suicide risk, technological failures, and increased emotional distance between the patient and provider, indicate a need to assess the effectiveness of different types of telehealth for different conditions [51]. This review contributes to a current understanding of the possibilities and challenges of various telehealth modalities but is itself limited. Our interest was in telehealth evidence over the past five years, and so we limited the scope of our review to that time period. Most of the articles in our review focused on the treatment of PTSD, with only a few articles addressing symptoms of depression, insomnia, and hazardous alcohol use, often in conjunction with PTSD. Overall, our review supports the non-inferiority of telehealth provided by VTC for PTSD and suggests benefit for internet-based self-guided therapy for PTSD, depression, and insomnia. Results for treatment of hazardous alcohol use indicated less effectiveness, although only five studies examined this diagnosis and only two focused solely on hazardous alcohol use. The usefulness of telehealth for other mental health conditions is not clear based on this review. The strength of the evidence for studies included in this review is mixed; many of the studies of VTC were well-conducted, large randomized controlled trials, while studies on internet-based therapy and mobile applications were a mix of prospective cohort and randomized controlled trials, some of which utilized waitlist controls.

This review focused on telehealth as a treatment modality for specific mental health conditions. Outside the scope of review, but also worthy of consideration, is the role of telehealth in facilitating engagement in mental health care. Stecker and colleagues showed that a single telemedicine CBT session aimed to modify beliefs about seeking treatment for PTSD decreased barriers to care and resulted in earlier engagement [52]. This is an important finding when considering the treatment dose required to show a positive result. In mental health, there are many important obstacles that patients face to include stigma, denial or lack of insight regarding the severity of the illness, access, ability to take time away from work, and beliefs about the impact of treatment on their career. The ability of telehealth to reduce these obstacles is a topic of ongoing study.

## Artificial Intelligence

Any paper discussing the future of health technology in 2025 is incomplete without a discussion of AI. The role of AI in mental health care is a topic worthy of its own review and is outside the scope of this paper. Interested readers can find reviews of early research on this topic in several recent papers [53–55]. Our team wondered how AI might contribute to the work of synthesizing medical literature to answer a clinical question. As a demonstration of the current state of the art, the authors manually synthesized the data for the articles in this review and then created a notebook in Google’s NotebookLM Plus™ (Mountain View, California, henceforth NotebookLM) using the same 30 articles to compare an AI platform’s synthesis of the information to ours.

We used the prompt: “Please use these sources to answer the question ‘What is the current evidence for benefit of tele-behavioral health treatment for military and veteran populations with Post-Traumatic Stress Disorder, Major Depressive Disorder, Anxiety, Insomnia, Substance Use Disorders or Adjustment Disorders?’ Please comment on different types of telehealth, what if any benefit was shown, and the strength of the evidence for that benefit.”

NotebookLM provided a detailed, lengthy answer which is available to readers in Supplemental Data. When asked to summarize this response in a paragraph of five sentences, NotebookLM responded: “Based on the provided sources and our conversation history, tele-behavioral health treatment demonstrates significant potential for military and veteran populations across various mental health conditions, largely by addressing barriers to traditional care. Videoconferencing stands out with strong evidence showing its effectiveness, particularly for PTSD, often demonstrating outcomes comparable to in-person therapy. Other modalities like web-based interventions and blended approaches combining online content with telehealth support have also shown promising results in reducing symptoms of PTSD, depression, and improving social functioning. Mobile applications offer preliminary evidence primarily as accessible self-management tools for conditions like insomnia, with some initial pilot findings for PTSD and hazardous alcohol use showing mixed or limited effects. Overall, the strength of the evidence varies by modality and condition, with the most robust findings supporting synchronous telehealth for PTSD and growing support for blended models and some web-based programs across related conditions, while evidence for stand-alone apps and for conditions like SUD and Adjustment Disorders remains more preliminary.”

As a team, we were interested to note that NotebookLM’s conclusion about the current evidence for telehealth benefit was similar to our own, although without the time we had invested in reviewing and synthesizing the literature prior to our query we would not have been able to check the accuracy of NotebookLM’s response. AI systems have significant limitations, including the potential to generate false answers that sound plausible, lack of transparency around how the AI generates responses, concerns for data ownership and the risk of perpetuation of bias [56]. NotebookLM strives to overcome some of these limitations through a source-grounded approach and through transparency in linking responses to source materials [57]. Nevertheless, any use of an AI system to synthesize literature to answer a clinical question should be conducted cautiously; clinicians need to verify the accuracy of information before implementing it with patients.

## Conclusions

Our review highlights both the promise of telemedicine initiatives but also the paucity of high-quality evidence comparing outcomes with traditional face-to-face treatments, particularly for internet-based and mobile application therapies. Within this review, a majority of the trials have come from the VA population, further reinforcing the need for the MHS to increase research on interventions that could benefit active duty servicemembers, who face different constraints and stressors than veterans. Despite current limitations, we conclude there is enough evidence to show that in the treatment of PTSD we must move away from the question of “does telemedicine work?” to the question “how can we make telemedicine most efficient and effective?” for veteran and ADSM populations. Other disorders still require research into the effectiveness of telehealth. The MHS and VHA have the infrastructure in place to continue studying telehealth and to rapidly scale effective telehealth solutions. Knowing which solutions are effective allows wise use of resources.

Our recommendations to advance our understanding of telehealth for mental health care in both the VHA and the MHS are to:

1. Increase investment in national centers for virtual mental health care and assign experts in mental health treatments to these facilities in a “hub and spoke” model. These specialists can utilize VTC, a telehealth model with strong evidence of benefit for PTSD, to expand access to care for Veterans and ADSMs across geographic distance and can accommodate after-hours care by taking advantage of different time zones. These national centers could support further high-quality research around the benefits of telehealth for the care of mental health disorders other than PTSD.
2. Conduct further randomized controlled trials with active controls and outcomes determined by blinded assessors on diagnoses other than PTSD to develop the evidence base for telehealth in mental health. Research on mobile applications, the type and timing of synchronous support for individuals using asynchronous internet-based telehealth, and comparisons between various telehealth approaches is needed to determine which treatments can have the greatest impact.
3. Create a centralized, government funded clearinghouse to assess which currently available internet-based programs and mobile applications are effective, and how they can best integrate into mental health treatment offered in each system. Providers and patients alike would benefit from clear information from a trusted source on which of many available options have evidence of benefit.
4. We recommend the MHS support increased research on interventions for ADSMs; these individuals represent a different population than Veterans and therefore findings specific to Veterans may not be fully transferable to the ADSM population.
5. Continue to study the role of artificial intelligence in synthesizing medical information and in supporting clinical care.

## Key References

- Heyworth L, Shah N, Galpin K. 20 Years of Telehealth in the Veterans Health Administration: Taking Stock of Our Past and Charting Our Future. J Gen Intern Med. 2024;39(Suppl 1):5–8. doi: 10.1007/s11606-024-08617-w.
  - ○ Provides a historical overview of telehealth in the Veterans Health Administration and plans for expansion.
- Madsen C, Poropatich R, Koehlmoos TP. Telehealth in the Military Health System: Impact, Obstacles, and Opportunities. Mil Med. 2023;188(Suppl 1):15–23. doi: 10.1093/milmed/usac207.
  - ○ Provides a historical overview of telehealth in the Military Health System and a snapshot of current programs, current opportunities, and current challenges.
- Morland LA, Mackintosh MA, Glassman LH, Wells SY, Thorp SR, Rauch SAM, et al. Home-based delivery of variable length prolonged exposure therapy: A comparison of clinical efficacy between service modalities. Depression and Anxiety. 2020;37(4):346 − 55. doi: 10.1002/da.22979.
  - ○ Comparison of prolonged exposure therapy delivered by home-based telemedicine, office-based telemedicine, and in-home in-person care; improvement in PTSD symptoms did not differ significantly between modalities although in-home in-person care had the highest retention rate.
- Peterson AL, Mintz J, Moring JC, Straud CL, Young-McCaughan S, McGeary CA, et al. In-office, in-home, and telehealth cognitive processing therapy for posttraumatic stress disorder in veterans: a randomized clinical trial. BMC Psychiatry. 2022;22(1):41. doi: 10.1186/s12888-022-03699-4.
  - ○ Comparison of cognitive proessing therapy delivered by home-based telemedicine, office based in-person care, and in-home in-person care; improvement in PTSD symptoms did not differ significantly between modalities.
- Cloitre M, Amspoker AB, Fletcher TL, Hogan JB, Jackson C, Jacobs A, et al. Comparing the ratio of therapist support to internet sessions in a blended therapy delivered to trauma-exposed veterans: Quasi-experimental comparison study. JMIR Mental Health. 2022;9(4):1–12. doi: 10.2196/33080.
  - ○ Compares different coaching frequences for an available internet-based self-guided therapy (webSTAIR: web-based Skills Training for Affective and Interpersonal Regulation) for PTSD and depression symptoms.
- Davis JP, Pedersen ER, Borsari B, Bowen S, Owen JE, Sedano A, et al. Effects of a Mobile Mindfulness Smartphone App on Posttraumatic Stress Disorder Symptoms and Alcohol Use Problems for Veterans: A Pilot Randomized Controlled Trial. Journal of Consulting and Clinical Psychology. 2025;93(2):96–109. doi: 10.1037/ccp0000940.
  - ○ A randomized controlled trial of a mindfulness mobile application based therapy for PTSD.

## Supplementary Information

Below is the link to the electronic supplementary material.

Supplementary File 1 (DOCX 12.1 KB)

## Data Availability

No datasets were generated or analysed during the current study.

## Declarations

### **Disclaimer**

The opinions and assertions expressed herein are those of the author(s) and do not reflect the official policy or position of the Uniformed Services University of the Health Sciences or the Department of Defense.

Generative Artificial Intelligence was used in the section of the paper titled“Artificial Intelligence” to synthesize findings from the literature as an example for readers of what this technology can accomplish. The authors independently completed a synthesis of findings prior to utilizing generative artificial intelligence.
