# Supplementary material for: Staying Relevant in the Digital Age: Exploring the Evolving Frontier of Telehealth for Mental Health in the Military Health System and Veterans Health Administration
Source: Curr Psychiatry Rep. 2025 Dec 29;28(1):8. doi: 10.1007/s11920-025-01651-3 (PMC12748128; doi:10.1007/s11920-025-01651-3)
Supplement: Supplementary file 1 — (DOCX 12.1 KB) [file 11920_2025_1651_MOESM1_ESM.docx]

Staying Relevant in the Digital Age: Exploring the Evolving Frontier of Telehealth for Mental Health in the Military Health System and Veterans Health Administration

Authors:

Elizabeth A. Greene MD; Uniformed Services University of the Health Sciences

Eric J. Serpico DO; Uniformed Services University of the Health Sciences

Gary L. Legault MD; Uniformed Services University of the Health Sciences

Scott G. Williams MD; Uniformed Services University of the Health Sciences

**Supplemental Data**

**Prompts and Responses from Notebook LM**

**8 June 2025**

Prompt 1: Please use these sources to answer the question "What is the current evidence for the benefit of tele-behavioral health treatment for military and veteran populations with Post-Traumatic Stress Disorder, Major Depressive Disorder, Anxiety, Insomnia, Substance Use Disorders, or Adjustment Disorders?" Please comment on different types of telehealth, what if any benefit was shown, and the strength of the evidence for that benefit.

Based on the provided sources and our conversation history, tele-behavioral health treatments show promise for military and veteran populations with various mental health conditions, often addressing significant barriers to traditional in-person care. Different modalities, including videoconferencing, web-based programs, mobile applications, and blended approaches, have been explored with varying levels of evidence supporting their benefit.

Here is a summary of the current evidence for specific conditions:

**Post-Traumatic Stress Disorder (PTSD)**

- **Videoconferencing (Telehealth/VC/Home-Based Telehealth [HBT]/Office-Based Telehealth [OBT]/Tele-therapy):**
  - **Benefit Shown:** Videoconferencing is a widely studied modality for delivering evidence-based psychotherapies (EBPs) for PTSD, such as Cognitive Processing Therapy (CPT) and Prolonged Exposure (PE). Studies show that delivering CPT and PE via videoconferencing can be **as effective as in-person delivery** in reducing PTSD symptoms. This includes individual and group formats. Telehealth can also improve **psychosocial functioning** and quality of life. A major benefit is addressing barriers to care like distance, travel time and cost, difficulty scheduling, and stigma concerns. HBT, where treatment is delivered to the veteran's home, eliminates more barriers than OBT (delivery to a local clinic). Some veterans prefer HBT for reasons like avoiding potential sexual harassment at VA facilities or having a more comfortable environment.
  - **Strength of Evidence:** The evidence for videoconferencing is **strong**, supported by multiple randomized controlled trials (RCTs) and non-inferiority trials. These studies often used rigorous designs, including non-inferiority analyses to compare efficacy to in-person treatment.
- **Web-based Interventions (Online/iCBT/eHealth):**
  - **Benefit Shown:** Web-based treatments for PTSD can lead to significant reductions in PTSD symptoms. They offer flexibility, anonymity, and cost-effectiveness compared to in-person therapy. Some studies show better outcomes when web interventions include therapist support compared to completely self-guided approaches. Web-based adapted EBPs like Written Exposure Therapy (WET) delivered online have shown effectiveness in improving PTSD symptoms, depressive symptoms, and functioning, particularly when facilitated by peer coaches. Web-PE, an online version of PE, significantly reduced PTSD symptoms in both an RCT (though with smaller effect size than in-person PCT) and an open trial (with a larger effect size). Web-based interventions like VetChange also address co-occurring issues like hazardous alcohol use.
  - **Strength of Evidence:** Evidence for web-based interventions alone is **more varied** than for videoconferencing, ranging from pilot studies and open trials to RCTs. While meta-analyses support the efficacy of iCBT for PTSD, effects can be moderate, and superiority over active controls is not always found.
- **Mobile Applications (mHealth):**
  - **Benefit Shown:** Mobile apps serve as accessible, on-demand self-management tools. PTSD Coach, a well-supported app, has shown potential for reducing PTSD symptoms in pilot studies, with one pilot RCT showing symptom reduction, although the effect was small or not statistically significant compared to waitlist. The Renew app, focusing on exposure therapy and social support, is being piloted. Mind Guide, a mindfulness-based app with alcohol content, showed significant reductions in PTSD symptoms in a pilot RCT, although it did not show a difference in alcohol use or consequences compared to the control.
  - **Strength of Evidence:** The evidence for stand-alone mobile apps is currently **preliminary**, primarily based on pilot studies and smaller RCTs. More research is needed to test the efficacy of apps specifically addressing PTSD symptoms.
- **Blended Therapy (Web-based + Telehealth Coaching):**
  - **Benefit Shown:** Blended approaches integrating web-based programs with therapist support via telehealth (e.g., webSTAIR) have shown **significant improvements in PTSD symptoms, depression, social functioning, emotion regulation, and interpersonal problems** in veterans. They combine the flexibility of online content with the clinical advantages of therapist interaction. Studies suggest that even a limited number of coaching sessions (e.g., 5 biweekly sessions) can be as effective as more frequent sessions (e.g., 10 weekly sessions). This modality is feasible, acceptable, and potentially efficient.
  - **Strength of Evidence:** Evidence for blended therapies is **promising and growing**, supported by RCTs and quasi-experimental comparison studies demonstrating effectiveness and non-inferiority of lower-intensity support models.

**Major Depressive Disorder (MDD)**

- **Benefit Shown:** Depression is often co-occurring with PTSD in veterans. Several tele-behavioral health interventions designed primarily for PTSD or other conditions have shown benefit for depressive symptoms as a secondary outcome. Videoconferencing delivery of CPT and PE can lead to reductions in depressive symptoms. However, one study found HBT PE to be inferior to in-person PE for depression outcomes at post-treatment and 3-month follow-up. V-CBT, a video-to-home CBT tailored for depression in Parkinson's disease veterans, significantly outperformed treatment as usual in reducing depression scores. Web-based interventions like VetChange and Vets Prevail have shown reductions in depression symptoms, though effect sizes can be small. Blended webSTAIR has consistently shown significant reductions in depression symptoms. Couple HOPES, an online couples intervention, also showed improvements in depressive symptoms for the veteran partner.
- **Strength of Evidence:** Evidence for tele-behavioral health specifically for MDD in veterans is often tied to interventions for co-occurring PTSD. Stand-alone interventions like V-CBT for specific veteran populations (e.g., with Parkinson's) show strong evidence from RCTs. For interventions targeting co-occurring PTSD and depression, the evidence strength mirrors that of the primary PTSD outcome studies (e.g., strong for videoconferencing of EBPs, promising for blended therapies, varied for web-based).

**Anxiety**

- **Benefit Shown:** Anxiety is also a common comorbidity with PTSD. Studies on tele-behavioral health for PTSD often report on anxiety symptoms as secondary outcomes. PE delivered via HBT has shown some reduction in anxiety symptoms. Web-based interventions and blended therapies addressing trauma-related difficulties, such as webSTAIR, include focus on emotion regulation, which is relevant to managing anxiety. Meta-analyses in broader populations suggest efficacy of internet CBT for anxiety. Couple HOPES showed improvements in related issues like argumentativeness.
- **Strength of Evidence:** Similar to MDD, the evidence for anxiety treatment via tele-behavioral health in veterans is largely derived from studies primarily targeting PTSD. Evidence for managing related symptoms like emotion dysregulation through blended therapies is promising. General evidence for internet CBT for anxiety is supported by meta-analyses.

**Insomnia**

- **Benefit Shown:** Insomnia is highly prevalent among veterans, often co-occurring with conditions like PTSD and depression. Cognitive Behavioral Therapy for Insomnia (CBT-I) is the recommended first-line treatment, but access is limited. Telehealth delivery to the home of group CBT-I shows promise for reducing insomnia severity with comparable effect sizes to in-person treatment, while being highly acceptable and reducing barriers. Internet-delivered CBT-I (iCBT-I) programs like SHUTi are effective in non-veteran populations. In veterans, use of a digital CBT-I program resulted in reduced insomnia and less medication use in an implementation study. However, an RCT of unguided SHUTi in veterans did not show greater improvement than an education website control. Mobile apps like Insomnia Coach and CBT-I Coach show feasibility, acceptability, and preliminary efficacy for reducing insomnia symptoms in pilot studies.
- **Strength of Evidence:** Evidence for telehealth delivery of CBT-I in veterans is **growing but still developing**. Pilot studies show promise for group telehealth and mobile apps. Evidence for unguided iCBT-I effectiveness in veterans is mixed based on the sources. Dissemination efforts within the VA system are increasing access to CBT-I via telehealth.

**Substance Use Disorders (SUD)**

- **Benefit Shown:** Hazardous alcohol use and AUD are common among veterans, often co-occurring with PTSD. Veterans often misuse alcohol to cope with PTSD symptoms. Tele-behavioral health interventions are being explored to address this comorbidity. The Mind Guide mobile app (mindfulness + alcohol) showed benefit for PTSD symptoms but no difference in alcohol use or consequences compared to a control. The VetChange web-based program (CBT + PTSD) showed a significant reduction in alcohol use over time, but this was linked to increased PTSD hyperarousal and higher dropout risk, highlighting the complex interplay between symptoms. Thinking Forward, a web-based CBT program, reduced heavy drinking in veterans with substance use and PTSD in an earlier trial. A pilot study combining Thinking Forward with peer support found improvements in quality of life and coping but no difference in outcomes between the peer-supported and self-managed versions. A computer-based intervention (SHE) for women veterans with sexual trauma and health risks including hazardous drinking showed feasibility and acceptability in a pilot.
- **Strength of Evidence:** Evidence for tele-behavioral health directly targeting SUD in veterans is **limited and preliminary**, often focusing on interventions for co-occurring PTSD and hazardous drinking. While some interventions show promise for reducing alcohol use, the complex relationship with PTSD symptoms and dropout needs further investigation. Evidence for specific modalities like mobile apps is preliminary.

**Adjustment Disorders**

- **Benefit Shown:** While adjustment disorders are mentioned in the context of VA/DoD clinical practice guidelines, the provided sources do not contain specific studies evaluating the benefit of tele-behavioral health interventions designed primarily for this condition in military or veteran populations.
- **Strength of Evidence:** There is **no direct evidence** for tele-behavioral health treatment of Adjustment Disorders in military and veteran populations presented in these sources.

Prompt 2: Can you summarize the above responses in a paragraph of 5 sentences?

Based on the provided sources and our conversation history, tele-behavioral health treatment demonstrates significant potential for military and veteran populations across various mental health conditions, largely by **addressing barriers to traditional care**. **Videoconferencing** stands out with **strong evidence** showing its effectiveness, particularly for **PTSD**, often demonstrating outcomes **comparable to in-person therapy**. Other modalities like **web-based interventions** and **blended approaches** combining online content with telehealth support have also shown **promising results** in reducing symptoms of PTSD, depression, and improving social functioning. **Mobile applications** offer preliminary evidence primarily as accessible **self-management tools** for conditions like insomnia, with some initial pilot findings for PTSD and hazardous alcohol use showing mixed or limited effects. Overall, the strength of the evidence varies by modality and condition, with the most robust findings supporting synchronous telehealth for PTSD and growing support for blended models and some web-based programs across related conditions, while evidence for stand-alone apps and for conditions like SUD and Adjustment Disorders remains more preliminary.
